# Investigation of characteristics and risk factors of sports injuries in young soccer players: a retrospective study

**DOI:** 10.1186/1755-7682-6-14

**Published:** 2013-04-20

**Authors:** Fabio Nascimento Bastos, Franciele Marques Vanderlei, Luiz Carlos Marques Vanderlei, Jayme Netto Júnior, Carlos Marcelo Pastre

**Affiliations:** 1 Departamento de Ciências Patológicas, UEL – Univ Estadual de Londrina, Londrina, PR, Brazil; 2 Rua Napoleão de Barros, 715 - Térreo, São Paulo, Brazil; 3 Faculdade de Ciências e Tecnologia. Programa de Pós-Graduação em Fisioterapia, Laboratório de Fisioterapia Desportiva – LAFIDE, Presidente Prudente, UNIFESP – Univ Federal de São Paulo, São Paulo, SP, Brazil

**Keywords:** Child, Adolescent, Athletic injuries, Risk factors

## Abstract

**Background:**

The participation of children and adolescents in sports has become increasingly frequent, including soccer. This growing involvement gives rise to concerns regarding the risk of sports injuries. Therefore, the aim of the present study was to describe the musculoskeletal injuries in young soccer players.

**Methods:**

301 male soccer players with a mean age 14.67 ± 2.08 years were randomly recruited. The Referred Condition Inquiry was used to collect information on the mechanism of injury and anatomic site affected as well as personal data on the participants. The variables were analyzed based on the degree of association using Goodman’s test for contrasts between multinomial populations, with the p < 0.05.

**Results:**

Among the 301 athletes, 24.25% reported at least one injury. With regard to height, taller individuals reported more injuries than shorter individuals (62.5% and 37.5%, respectively; p < 0.05). Injuries were more frequent among players with a training duration greater than five years (69.65%) in comparison to those who trained for a shorter duration (30.35%) (p < 0.05). The lower limbs, especially the ankle/foot and knee, were the most affected anatomic sites. Impact was the most common mechanism of injury.

**Conclusion:**

The young practitioners of soccer analyzed had low rates of injury. The main causal mechanism was the impact. A taller height and longer exposure to training were the main risk factors for injury among young soccer players.

## Background

Soccer mobilizes millions of fans in several countries and the search for his practice covers different social strata and age groups, including children and adolescents, due mainly to the perception on the part of parents that the sport is safe [1-3]. However, recent studies have identified a high risk of injury. While it has been well established that the frequency and severity of injury among adults in striking in comparison to other sport modalities, there has been little investigation into soccer injuries among young athletes [1,4].

There is a consensus that the practice of sports offers numerous benefits [4,5]. However, constant exposure to repetitive athletic actions and overload place the integrity of bodily structures at risk [6], especially in cases in which growth and maturation are not yet completely developed, such as in childhood and adolescence. In such cases, intensive, specific demands in sports are a potential risk for the occurrence of injuries [7,8].

Besides the growing popularization of the practice of soccer at more advanced performance levels among youths, the motor, technical and tactical demands of training and competition lead to greater exposure to causal factors of injury [9]. The amount of practice time, nature of participation, growth phase and biological maturity have been suggested as possible contributing factors to the occurrence of injury among children and adolescents. It therefore seems appropriate to investigate the causal relations of sports injuries with the aim of minimizing the risk and adopting appropriate preventive measures specific to each case. The aim of the present study was to describe the musculoskeletal injuries in young soccer players.

## Methods

### Subjects

The sample was made up of 301 soccer athletes belonging to sports initiation schools organized by the Secretaria Municipal de Esportes de Presidente Prudente - SEMEPP (Presidente Prudente, São Paulo, Brazil). The participants were all male and recruited randomly, with a mean age of 14.67 ± 2.08 years, weight of 58.62 ± 12.98 kg, height of 1.65 ± 0.12 m, body mass index (BMI) of 21.23 ± 3.28 kg · m^-2^ and training duration of 4.86 ± 2.74 years and trained on average three times a week lasting one hour per day. Athletes whose legal guardians did not sign the term of informed consent and those having practiced the sport for less than one year were excluded. The study received approved by the Research Ethics Committee of the School of Science and Technology of the Universidade Estadual Paulista (Presidente Prudente, SP, Brazil).

### Study design and field procedures

A retrospective study was carried out. The data were collected through individual interviews, performed by a single interviewer, using a reported condition inquiry addressing the occurrence of injury and its characteristics in the current season (previous 12 months of training and/or competition). The volunteers were approached either prior to or following training sessions in order not to interfere in the normal dynamics and routine of the sport.

The data were collected using the Referred Condition Inquiry (Table 1) in the period from May to August 2011. Inquiries are the most appropriate form of obtaining information on the health status of specific population groups [10]. A pilot study was first conducted to adjust the data acquisition procedures and test the inquiry on a population with similar characteristics to those of the present study, which confirmed the full possibility of use and fit in the proposed methodological design.

**Table 1:** Reported condition inquiry used to collect data on sports injury

**V0-Identification:**													
**V1-Age:**													
**V2-Weight:**													
**V3-Height:**													
**V4-Training time:**													
**V5-Injury:**													
**VARIABLE**	**INJURIES**												
	1	2	3	4	5	6	7	8	9	10	11	12	13
**V6**-**Anatomical site**													
**V7-Mechanism of injury**													
**V4-Training time**	**V5-Injury**			**V6-Anatomical Site**					**V7-Mechanism of Injury**				
Noted in years	0 No			1 Neck					1 Running				
	1 Yes			2 Shoulder					2 Jump				
				3 Arm					3 Impact				
				4 Dorsal					4 Specific Athletic Gesture				
				5 Elbow									
				6 Forearm									
				7 Lumbar Region									
				8 Wrist/Hand									
				9 Hip/Thigh									
				10 Knee									
				11 Calf									
				12 Ankle/Foot									

Comments: V7–regarding specific athletic gesture (7), to note which gesture.

The interviews were carried out by a single interviewer familiarized with the instrument. Following the presentation of the objectives and legal terms for participation in the study, the soccer players responded to the questions posed by the researcher, who was also responsible for recording the answers on the inquiry in an individual fashion. Pastre et al., 2004 [10] suggests this procedure based on different degrees of understanding regarding the annotation of answers on the part of interviewees. The forms were then numbered in order to facilitate the recording of the information and the data were recorded on computational spreadsheets for organization, systematization and analysis.

### Description of reported condition inquiry

The Referred Condition Inquiry was adapted from previous studies [10,11] and contained personal data on the athletes: age, weight, height and duration of training. The questionnaire also contained items on the mechanism of injury and affected anatomic site. An illustration of the human body was shown to the volunteer in order to facilitate the identification of the anatomic site of the injury and the subject marked the region of the body referring to the sensation of pain or musculoskeletal discomfort. The determination of the injury mechanism consisted of the volunteer’s perception regarding the contact or exact action performed when signs and symptoms of an acute episode emerged and/or the type of activity in which such manifestations were accentuated.

This questionnaire is reliable for recording information regarding sports injuries within a complete season of training and competition [10]. For the purposes of the present study, a sports injury was considered any injury of the musculoskeletal system, the signs and symptoms of which originated from the practice of the sport either during training or competition that compromised normal training in terms of form, duration, intensity or frequency [10,11].

### Organization and description of categories of variables

In order to facilitate the analysis and presentation of the results, the categories or subdivisions of the variables were grouped into more expressive blocks of results without altering the essence of their origin or conclusions of the study. Regarding anatomic site of pain or discomfort, the questionnaire listed 20 bodily regions, which were grouped in the following segments: upper limbs, trunk, thigh/leg, knee and foot/ankle.

The mechanism of injury was highlighted, which was the perception of the athlete regarding the exact moment in which typical signs and symptoms are accentuated, such as running, jumping, impact (direct contact, collision with opponent) and specific action (change in direction, kicking, dribbling, heading).

### Statistical analysis

Descriptive statistics were performed through central tendency and variability (mean and standard deviation). Normality of the data was determined using the Kolmogorov-Smirnov test. The association of the quantitative variables was analyzed in relation to the presence or absence of injury. Variables with parametric distribution were analyzed using the Student’s *t*-test and non-parametric variables were analyzed using the Mann–Whitney test. The Odds Ratio and 95% confidence intervals were determined for the anthropometric and training variables that demonstrated significant differences in the comparison between the presence and absence of injury. Associations between the qualitative variables involving characteristics of the injuries and sport were determined using Goodman’s test for contrasts between and within multinomial populations. The level of significance was set at 5% for all statistical tests.

## Results

Among the 301 athletes, 73 (24.25%) reported at least one injury in the previous 12 months (0.24 injury per athlete and 1.24 per injured athlete). No statistically significant differences were detected with regard to age, weight and BMI in the comparison between groups with and without injury (p > 0.05) (Table 2).

**Table 2:** Descriptive measures of anthropometric variables and training according to the presence or absence of injury

**Variable**	**Injury**	
	**Presence (n = 56)**	**Absence (n = 245)**
Age (years)	14.91 ± 1.95	14.61 ± 2.11
Weight (Kg)	61.46 ± 12.52	57.97 ± 13.02
Height (m)	1.69 ± 0.11*	1.64 ± 0.12
Body mass index (Kg.m^-2^)	21.34 ± 2.67	21.21 ± 3.40
Duration of training (years)	5.84 ± 2.86*	4.63 ± 2.67

Values are means ± standard deviation.

* Indicate p < 0.05 for presence versus absence injury.

Players taller than 1.67 m reported more injuries than those with 1.66 m or shorter (p = 0.01) (Table 3). Players with more than five years of training reported more injuries than those with less than five years of training (p = 0.003) (Table 3).

**Table 3:** Absolute and relative distribution of for the presence or absence of injury according to height and duration of training

**Variable**	**Injury**	
	**Presence (n = 56)**	**Absence (n = 245)**
Short ^1^	21 (37.5%)	130 (53.06%)
Tall ^2^	35 (62.5%)	115 (46.94%)
Shorter ^3^	17 (30.35%)	122 (49.80%)
Longer ^4^	39 (69.65%)	123 (50.20%)

Sample characterization: 1.69 ± 0.11 m [1.67 – 1.72].

OD: 1.88 IC 95%: 1.04-3.42.

Sample characterization: 5.84 ± 2.86 years [5.44 – 6.78].

OD: 2.28 IC 95%: 1.22-4.24.

^1^ cutoff point of lower bound of confidence interval (< 1.67 m) obtained from participants for height variable.

^2^ cutoff point for upper bound of confidence interval (≥ 1.67 m) obtained from participants for height variable.

^3^ cutoff point of lower bound of confidence interval (< 5 years) obtained from participants for duration of training.

^4^ cutoff point of upper bound of confidence interval (≥ 5 years) obtained from participants for duration of training.

Shorter players reported a greater frequency of ankle/foot injuries in comparison to the upper limbs, trunk and thigh/leg. Taller athletes reported a greater frequency of knee and foot/ankle injuries in comparison to the trunk. There were no statistically significant differences within each height group (p > 0.05). Regarding duration of training, athletes with fewer years of training reported a greater frequency of injury in the ankle/foot in comparison to the upper limbs, trunk and thigh/leg, whereas athletes with more years of training reported a greater frequency of injury in the ankle/foot and knee in comparison to the upper limbs and trunk. There were no statistically significant differences between those who trained longer and those who trained for a shorter period of time. These results are displayed in Table 4.

**Table 4:** Absolute and relative (%) distribution of height and duration of training according to anatomic site affected

**Variable**	**Characteristic**	**Anatomic site**				
		**Upper limbs**	**Trunk**	**Thigh/leg**	**Knee**	**Foot/ankle**
Height (m)	Short ^1^	1(4.3)	0(0.0)	3(13.0)	5(21.7)	14(61.0)*
	Tall ^2^	6(12.0)	2(4.0)	8(16.0)	14(28.0)#	20(40.0)#
Duration of training (years)	Shorter ^3^	1(5.3)	0(0.0)	1(5.3)	6(31.6)	11(57.8)*
	Longer ^4^	6(11.1)	2(3.7)	10(18.5)	13(24.1)#	23(42.6)#

^1^ cutoff point of lower bound of confidence interval (< 1.67 m) obtained from participants for height variable; ^2^ cutoff point for upper bound of confidence interval (≥ 1.67 m) obtained from participants for height variable; ^3^ cutoff point of lower bound of confidence interval (< 5 years) obtained from participants for duration of training; ^4^ cutoff point of upper bound of confidence interval (≥ 5 years) obtained from participants for duration of training; Goodman’s test for contrasts between and within multinomial populations; values between parentheses are relative frequencies. * Statistically significant differences in relation to upper limbs, trunk and thigh/leg. # Statistically significant differences in relation to upper limbs and trunk.

Regarding the mechanism of injury (Table 5), impact was the main cause of injury among shorter athletes in comparison to running, jumping and specific action. In the comparison between shorter and taller athletes, statistically significant differences were found for the mechanisms of jumping and impact; taller athletes were injured more from jumping than shorter athletes, whereas shorter athletes were injured more from impacts than taller athletes. Regarding duration of training (Table 5), soccer players with less training time reported a greater frequency of injuries stemming from impacts in comparison to running and jumping. There were no statistically significant differences between those who trained longer and those who trained for a shorter period of time in relation to mechanism of injury.

**Table 5:** Absolute and relative (%) distribution of height and duration of training according to mechanism of injury

**Variable**	**Characteristic**	**Mechanism of injury**			
		**Running**	**Jumping**	**Impact**	**Specific action**
Height (m)	Short ^1^	4(17.4)	0(0.0)	15(65.2) *‡	4(17.4)
	Tall ^2^	15(30.0)	8(16.0) †	17(34.0)	10(20.0)
Duration of training (years)	Shorter ^3^	2(10.5)	1(5.3)	12(63.1)#	4(21.1)
	Longer ^4^	17(31.5)	7(13.0)	20(37.0)	10(18.5)

^1^ cutoff point of lower bound of confidence interval (< 1.67 m) obtained from participants for height variable; ^2^ cutoff point for upper bound of confidence interval (≥ 1.67 m) obtained from participants for height variable; ^3^ cutoff point of lower bound of confidence interval (< 5 years) obtained from participants for duration of training; ^4^ cutoff point of upper bound of confidence interval (≥ 5 years) obtained from participants for duration of training; Goodman’s test for contrasts between and within multinomial populations; values between parentheses are relative frequencies. * Statistically significant differences in relation to running, jumping and specific action. # Statistically significant differences in relation to running and jumping. † Statistically significant differences in relation to short. ‡ Statistically significant differences in relation to tall.

## Discussion

This study aimed to describe the musculoskeletal injuries in young soccer players from the application of reported condition inquiry. It was observed that adolescent soccer practitioners have a high rate of lower limb injuries, predominantly in the knees and ankle/foot. Taller athletes and those with a longer training duration have a greater frequency of injury. The impact component seems to be the main causal mechanism for the occurrence of sports injuries among young practitioners of soccer.

The rate of sports injury in the present survey was 0.24 per player and 1.24 per injured athlete, which corroborates the concept put forth by Fhoholdt et al., 2009 [1] that soccer is a sport with a low risk of injury when children are analyzed. However, Yard et al., 2008 [12] report high rates of injury among schoolchildren when analyzing training and/or competition. These divergences may not be related to the demands of the sport, but rather to the physical constitution of the different samples. The development phase of this population is an important aspect, as incomplete growth and maturation are risk factors for the occurrence of injury [7,8]. Moreover, investigations into sports injuries differ with regard to the methods employed and involve subjects from different countries, which hinders comparisons between studies.

Regarding the frequency of injuries, taller players had higher rates of sports injury. Such athletes preferentially play physically demanding defensive positions [13], often running at full speed, with abrupt changes in direction. Moreover, the current style of soccer demands explosive force and intense acceleration, which may further predispose these players to injury [14].

Another risk factor for the occurrence of injury was the duration of training. Athletes with a greater number of years of training had higher rates of injury than those with fewer years of training. Caine et al., 2008 [4] report that greater exposure to training and competition is progressive over the years and that age is another important factor to the occurrence of injury, although no difference was detected with regard to the latter variable in the present study. We believe that in this study, data analysis for the occurrence of injury may have influenced the findings since it was not made an association of musculoskeletal affections with the hours of exposure player.

Still in relation to age, in a study involving 1272 young soccer players, Sullivan et al., 1980 [15] found that players under 10 years of age were rarely affected by injury (less than 1 injury per 100 participants), whereas the rate of injury among players above this age is reported to be 7.7 per 100 players, which demonstrates that greater exposure to training and competition leads to a greater risk of injury due to the high intensity of the activities [16]. Moreover, the games become more competitive with age and more years of training, which may explain why athletes with greater exposure to the sport become injured more than those with lesser training time [17].

The results of the present study corroborate those found in the literature regarding the anatomic site of sports injuries, as the lower limbs, specifically the knee and ankle/foot, were the most affected sites [9,18]. This is likely explained by the fact that soccer requires the predominant use of the lower limbs. The biomechanics of the knee favors ligament injuries, which occur due to excessive movements of rotation and anterior shift of the tibia in relation to the femur, characterized in soccer as deceleration, a change in direction and the foot planted firmly on the ground [18]. The vulnerability of the ankle/foot is related to its proximity to the ball, which is the main point of contact between practitioners [19].

With regard to the causal mechanism, shorter players were injured more from impacts than taller players. This may stem from the current change in soccer style, in which shorter players generally take up offensive positions and have more direct contact with the defenders of the opposing team [9]. According to Zanuto et al., 2010 [13], shorter individuals are responsible for moving the ball down field due to their greater agility. Physical contact through intensive and often violent checking predisposes these athletes to injuries inherent to soccer [20]. Moreover, studies claim that, together with the impact mechanism, soccer players have a high rate of injury through the indirect mechanism stemming from running and abrupt changes in direction [14,21].

While the main injury mechanism among taller athletes was impact as well, there was a greater frequency of injury stemming from jumping among these players in comparison to shorter players. Paterson A, 2009 [18] demonstrated that, when jumping, the movements of the knee correspond to planting the foot on the ground in varus and internal rotation or in valgus and external rotation. However, in soccer, the extensor and flexor muscles of the lower limbs take on extreme importance to stabilization, with a tendency toward a preference of the extensors over the flexors, which generally reflects in a significant force deficit among the flexors, making them more vulnerable, especially during a jump [1]. It has been hypothesized that the increase in the varus/valgus angle, together with instability and an increase in proximal force in the shearing of the tibia may be a determinant for taller athletes having a greater rate of injury with regard to this variable [22].

It is worth mentioning that the whole discussion is based only on inference from the position of the player with the stature and the occurrence of injury, and it is not an association between cause and effect. Thus, it opens a gap in knowledge to enable more robust investigation into this event.

The interest in practicing soccer begins at an increasingly earlier age, which has been accompanied by an increase in interest regarding sports injuries [1]. The present study addresses the real situation of the sport practiced most by young Brazilians and demonstrates that height and duration of training are risk factors for the occurrence of injury. The perspective to consider the association with risk factors for injury may provide an element of interest for discussion about associations between injury and its cause, especially regarding the training time of young players that constituted a danger to the occurrence of injury when early sports specialization occurs. From the observation of the actual presence of soccer injuries in young people and their association with the factors described in this study, we can propose the implementation of programs of information and guidance on the subject to all involved with this practice.

A number of limitations of the present study should be addressed. The investigation did not analyze tendencies in relation to the time athletes spend away from the sport due to injury or the severity of injuries – factors which could be useful to assessing injury patterns. Moreover, there was no separation of the players based on tactical position, only inferences on height based on previous studies. Another limitation to be pointed out is that the survey used to collect information about injuries in soccer is a strategically suitable model, however, not validated. Despite the simplicity of your questions, additional information on the respondents´ interpretation about the issues can provide greater certainty to the validity of the response and, thus, allow more precise inferences. It is also suggested an interviewer’s previous training and familiarization with the technical terms used in the sport. Moreover, although Pastre et al., 2004 [10] suggest a good correlation between the occurrence of injuries and their reports on a range of time, some information may be forgotten and, in this sense, the very design of the study limits the accurate reading on all the actual occurrences. However, the present investigation offers knowledge on the frequency of sports injuries that affect children and adolescents. This is a field of interest in current research and can help coaches, parents and the players themselves in the establishing strategies aimed at preventing injuries.

Joint actions uniting health and sports sciences, with the inclusion of physiotherapists in field, should be encouraged for the establishment of strategies aimed at offering greater safety to beginners in the practice of any sports modality. Actions of this nature may have a positive impact on health, especially among children and adolescent, as well as consequences in the social realm.

## Conclusion

Adolescent soccer practitioners have a high rate of lower limb injuries, predominantly in the knees and ankle/foot. Taller athletes and those with a longer training duration have a greater frequency of injury. The impact component seems to be the main causal mechanism for the occurrence of sports injuries among young practitioners of soccer.

## Consent

Written informed consent was obtained from the patient and from parents, guardians, or next in keen for publication of this report and any accompanying images.

## Competing interests

The authors declare that they have no competing interests.

## Authors’ contributions

FNB, CMP and FMV conceived of the study, participated in its design and coordination and helped to draft the manuscript. FNB, CMP, FMV, LCMV, and JNJ performed the statistical analysis and interpretation of data and prepared the draft manuscript. All authors participated in the design of the study and in critical review of the manuscript. All authors read and approved the final manuscript.
